# Regional Cardiac Dysfunction and Dyssynchrony in a Murine Model of Afterload Stress

**DOI:** 10.1371/journal.pone.0059915

**Published:** 2013-04-01

**Authors:** Michael Bauer, Susan Cheng, Kazumasa Unno, Fen-Chiung Lin, Ronglih Liao

**Affiliations:** 1 Cardiovascular Division, Department of Medicine, Brigham and Women’s Hospital, Harvard Medical School, Boston, Massachusetts, United States of America; 2 Chang Gung Memorial Hospital, Linkou, Taiwan; 3 Taipei Medical University Postgraduate Institute of Clinical Medicine, Taipei, Taiwan; Harvard Medical School, United States of America

## Abstract

Small animal models of afterload stress have contributed much to our present understanding of the progression from hypertension to heart failure. High-sensitivity methods for phenotyping cardiac function *in vivo*, particular in the setting of compensated cardiac hypertrophy, may add new information regarding alterations in cardiac performance that can occur even during the earliest stages of exposure to pressure overload. We have developed an echocardiographic analytical method, based on speckle-tracking-based strain analyses, and used this tool to rapidly phenotype cardiac changes resulting from afterload stress in a small animal model. Adult mice were subjected to ascending aortic constriction, with and without subsequent reversal of the pressure gradient. In this model of compensated hypertrophic cardiac remodeling, conventional echocardiographic measurements did not detect changes in left ventricular (LV) function at the early time points examined. Strain analyses, however, revealed a decrement in basal longitudinal myofiber shortening that was induced by aortic constriction and improved following relief of the pressure gradient. Furthermore, we observed that pressure overload resulted in LV segmental dyssynchrony that was attenuated with return of the afterload to baseline levels. Herein, we describe the use of echocardiographic strain analyses for cardiac phenotyping in a mouse model of pressure overload. This method provides evidence of dyssynchrony and regional myocardial dysfunction that occurs early with compensatory hypertrophy, and improves following relief of aortic constriction. Importantly, these findings illustrate the utility of a rapid, non-invasive method for characterizing early cardiac dysfunction, not detectable by conventional echocardiography, following afterload stress.

## Introduction

Chronic pressure overload is believed to result in global cardiac stress, primarily leading to compensated concentric hypertrophy and subsequently decompensating to contractile failure. [1] Small animal models of aortic constriction have provided a reliable model of pressure overload and have contributed much to our present day understanding of the pathophysiology underlying hypertensive heart disease, cardiac hypertrophy, and heart failure. Recent work in humans suggests that cardiac dysfunction may begin early in the course of exposure to increased afterload. [2] Detecting these early functional changes has been made possible by recent advances in the development of non-invasive imaging modalities that permit high-sensitivity evaluations of myocardial mechanical function. These advances include strain-based imaging for assessing dynamic cardiac performance across myocardial tissue regions and in multiple planes. [3] We and others have reported on the utility of strain-based imaging to reliably detect early changes in regional myocardial dysfunction and dyssynchrony in small animal models of myocardial infarction.[4]–[7] The potential utility of speckle-tracking-based strain analyses for evaluating cardiac performance in the setting of pressure overload in animal models is less clear. In this study, we used strain-based imaging in conjunction with a newly developed image-based algorithm to comprehensively identify the changes in global and regional myocardial function that occur in an experimental model of pressure overload induced by ascending aortic constriction and release. Using these methods, we observe experimental evidence of dyssynchrony and regional myocardial dysfunction occurring early, along with compensatory hypertrophy, which improves following relief of aortic constriction; these functional alterations are not detectable using conventional echocardiographic methods. Importantly, these findings illustrate the utility of a rapid, non-invasive echocardiographic method for characterizing early cardiac dysfunction in mouse models of pressure overload and cardiac hypertrophy.

## Materials and Methods

### Experimental Protocol

Adult C57/BL6 mice were obtained from Jackson Laboratories. Animals were housed in a temperature controlled facility with a 12 hour alternating day/night cycle and allowed free access to food and water. All animal procedures were approved by the Harvard Medical Area standing Institutional Animal Care and Use Committee (HMA IACUC; Assurance #A3431-01). Male mice (average body weight is 25±3 g) underwent either sham operation or ascending aortic constriction as described previously. [8] Briefly, animals were anesthetized using pentobarbital (65 mg/kg i.p.), intubated, and ventilated using a small animal respirator (Harvard Apparatus). For each animal, the chest was opened and the aortic arch visualized. A 26G needle was placed adjacent to the aorta, and a suture was tied around both the needle and aorta. The needle was then removed, leaving a 26G opening in the aorta and a resultant significant stenosis. Finally, the chest was closed and the animal was weaned off the respirator as soon as spontaneous breathing had stabilized. To ensure a similar pressure gradient was established in animal undergoing aortic constriction, the aortic stenosis gradient was measured using continuous wave Doppler echocardiography immediately following surgery (please refer to Supplemental Methods for detail procedures in **Supporting Information S1**). The mean aortic pressure gradient for all animals used in this study was 51±1 mmHg.

**Figure 1:**
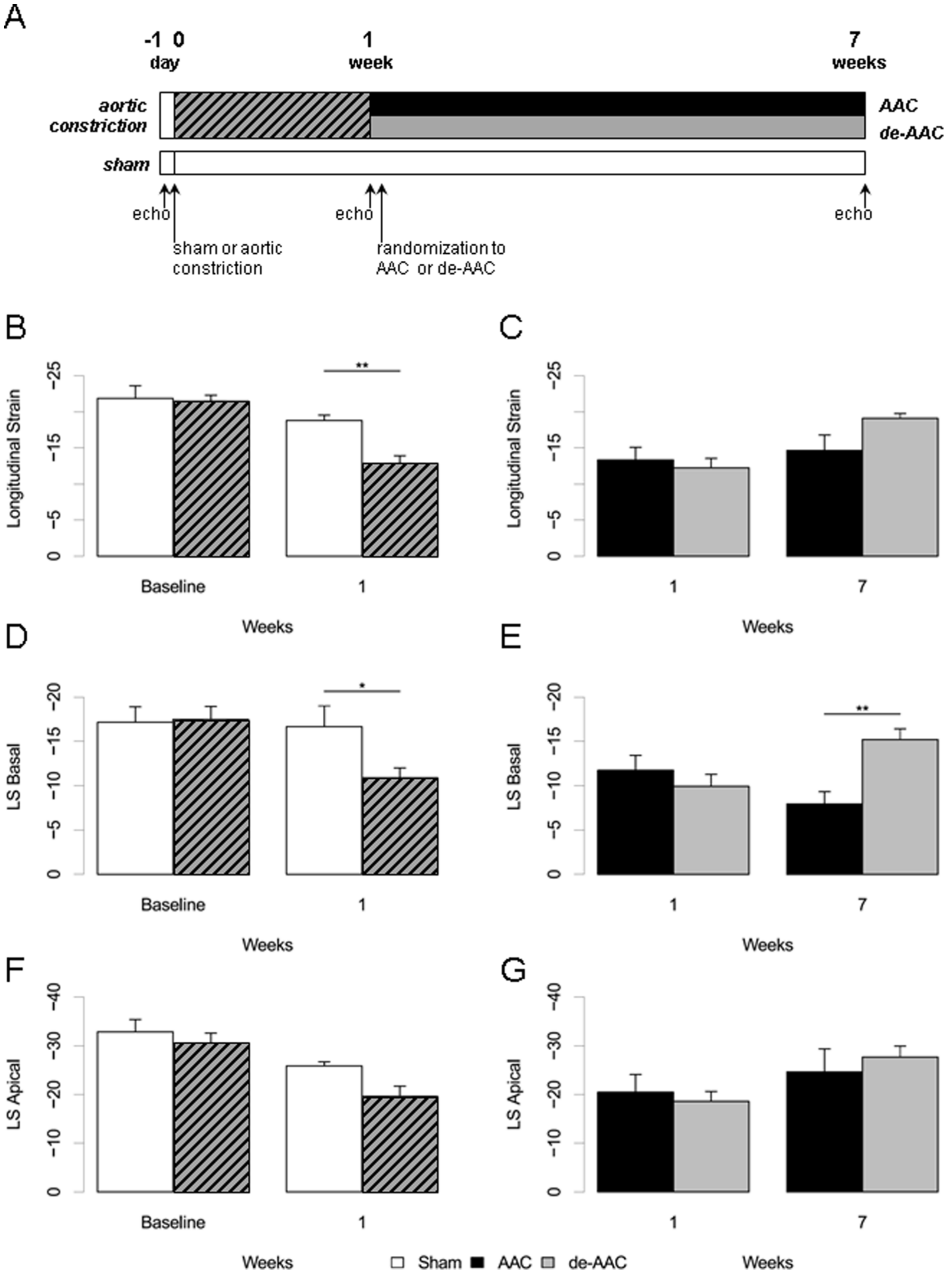
Changes in global and regional contractile function in an experimental model of pressure overload. The schematic of the experimental design is shown (**A**). Global peak longitudinal strain (endocardial) in the sham and aortic constricted animals at 1 week before randomization (**B**) and in the AAC and de-AAC animals at 7 weeks (**C**). Basal peak longitudinal strain (endocardial) in the sham and aortic constricted animals at 1 week (**D**) and in the AAC and de-AAC animals at 7 weeks (**E**). Apical peak longitudinal strain changes were similar to global peak longitudinal strain changes observed at 1 and 7 weeks (**F** and **G**).

One week post surgery, animals that received ascending aortic constriction were randomized into two groups, AAC and de-AAC groups (**Figure 1A**). The AAC group received another open chest operation during which the aorta remained banded (AAC group). In the de-AAC group, aortic constriction was released by removing the suture constricting the aorta.

### Echocardiography

Echocardiography was performed on conscious mice using a 28 MHz linear array transducer connected to a digital ultrasound console (Vevo2100 Visualsonics, Toronto, ON). All echocardiograms were performed at baseline (1 day before initial surgery), 1 week following initial surgery (before randomization into AAC versus de-AAC groups), and at 7 weeks. The echocardiography protocol is detailed in the **Supporting Information** **S1** section.

### Strain Analyses

Myocardial strain was measured from parasternal long-axis images using speckle-tracking-based imaging as described previously. [4] Briefly, acquired B-mode loops were imported into the VevoStrain software (VisualSonics, Toronto, ON). Three consecutive cardiac cycles were selected and the endocardium traced. Upon adequate tracing of the endocardium, an epicardial trace was added. Post-processed strain data were exported using the full data export option.

### Strain Topographical Mapping

Speckle-tracking-based strain allowed assessment of strains specific to 6 myocardial segments per LV view (**Figure S1** in **Supporting Information S1**). Internally, 8 points were measured for each of the 6 segments, resulting in 48 data points total. Topographical maps of longitudinal strain were produced using the Processing.org framework. To display localized strain values, location of the measured data points for both endocardial and epicardial tracings and strain values at these points were extracted from full data exports. The points were then plotted in their position in the original tracing, with the strain values color-coded for both endocardial and epicardial strain.

### Dyssynchrony Measurements

Ventricular dyssynchrony was determined from longitudinal strain using 3 separate methods: (i) maximum time-to-peak delay between the earliest and the latest segment; [3] (ii) time-to peak variation, defined as the standard-deviation of time-to-peak over all 6 segments; [3] and, (iii) a novel vector-based method. For the vector-based method, each of the 48 measured points from each strain curve was transformed into an n-dimensional vector with each dimension representing 1 frame. The strain value of each point at the specific frame was represented as the extension along the dimension. Angles between vectors using the inner vector products (θ = arccos((a·b)/|a||b|) were calculated as a measure of distance between all vectors, and mean vector angle was calculated as a measure of dyssynchrony. This third method for quantifying dyssynchrony was validated using a standard murine model of myocardial infarction (**Figure S2** in **Supporting Information S1**). A software application developed to calculate the novel dyssynchrony measurements is provided by the authors online at http://dyssynchrony.tentacleriot.eu/for the research community to use freely.

### Data Processing and Statistics

Peak global and segmental strain values and time-to-peak values were extracted from full data exports using a custom-written parser. Strain angles were calculated on top of this parser using Python and the Numpy package for linear algebra. Statistical analyses were performed using R (R 2.10.1). ANOVA tests, corrected for multiple measurements, were used to compare the groups across all time points. In cases where there was a significant between-groups difference, post-hoc t-tests with a Bonferroni-Holm correction for multiple testing were applied. A two-sided P value of <0.05 was considered the threshold for statistical significance.

## Results

A typical hypertrophic response was observed in mice subjected to aortic constriction within 1 week; this typical response was characterized by increased echocardiographic measures of LV wall thickness and calculated cardiac mass (**Table S1** in **Supporting Information S1**). Following de-AAC, pressure overload induced reactive hypertrophy improved within 6 weeks (**Table S1** in **Supporting Information S1**). However, no significant differences were observed in conventional echocardiographic measures of cardiac function at either earlier or later time points in the AAC compared with the sham-operated or de-AAC animals (**Table S1** in **Supporting Information S1**).

### Global and Regional Contractile Function

Despite the lack of difference observed in conventional fractional shortening measures, global peak longitudinal strain, representing a measure of overall myocardial contractile function, was significantly reduced in the AAC relative to the sham animals as early as within 1 week (**Figure 1B**). Reversal of aortic constriction resulted in slight improvement in peak global longitudinal strain in the de-AAC group compared to the AAC group (**Figure 1C**). Regional analyses of contractile function revealed more substantial differences in peak longitudinal strain between the AAC and sham animals at 1 week in the basal region (**Figure 1D**), and between the AAC and de-AAC animals at 7 weeks (**Figure 1E**). Apical peak longitudinal strain, however, was not altered between the AAC and sham animals (**Figure 1F**) or between the AAC and de-AAC animals (**Figure 1G**), suggesting localized effects of pressure overload on contractile function. Changes in apical strain were observed across time in all 3 groups of animals, but these changes were not significantly different between groups, suggesting an effect of surgery (including sham surgery) rather afterload stress and its reversal.

### Global and Regional Segmental Dyssynchrony

As shown in the cine loops available in the **Online Video S1**, topographical mapping of both endocardial and epicardial longitudinal strain across the long-axis regions of the LV offers the opportunity to track changes in strain throughout the heart cycle. In this video, the color scale is used to represent the spectrum of longitudinal strain values, where blue represents negative strain values and red represents positive strain values. In the sham-operated animals, clear apical predominance of peak endocardial strain was observed at 7 weeks. The AAC animals demonstrated increased wall thickness and preserved apical contractility. Interestingly, pressure overload and ventricular hypertrophy resulted in overt dyssynchrony at 7 weeks. Clustering of positive strain values were observed throughout contraction in both the endocardium and epicardium. In contrast, the de-AAC animals demonstrated decreased wall thicknesses and preserved segmental synchrony at 7 weeks (**Online Video S1**).

**Figure 2:**
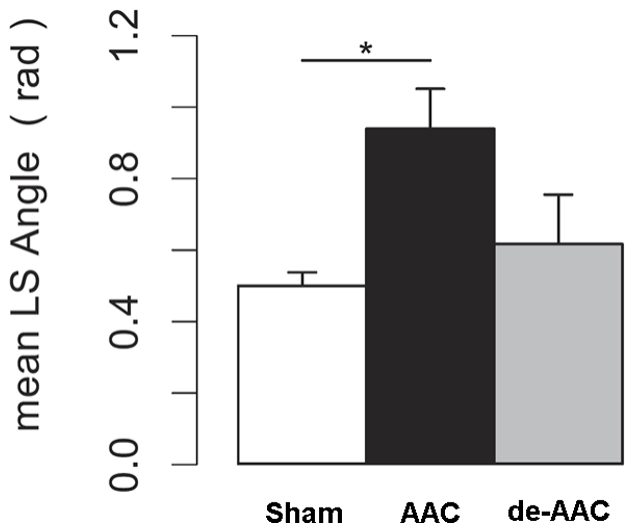
Vector angle dyssynchrony differentiates varying levels of exposure to afterload stress. Dyssynchrony measured as the mean vector angle (for 48 regional curves) for sham, AAC, and de-AAC animals at 7 weeks.

**Figure 3:**
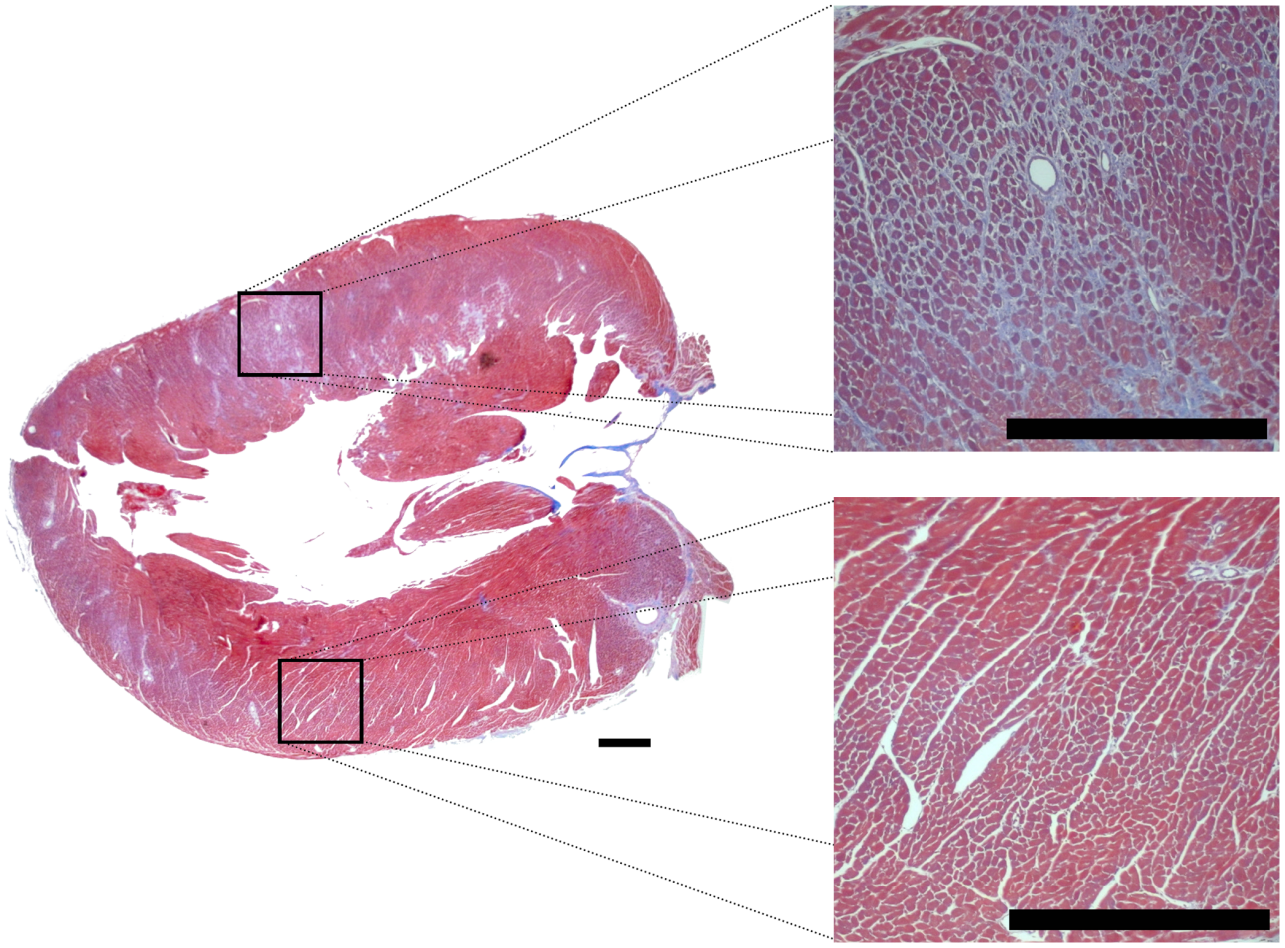
Regional distribution of fibrosis in a model of afterload stress. A Masson’s trichrome stained longitudinal section of the left ventricle of a representative mouse that underwent aortic banding (without reversal) is shown. Inner sections show regional variation in the degree of interstitial fibrosis across the left ventricular myocardium. The black horizontal scale bars represent 50 micrometers.

Three methods were employed to assess dyssynchrony in contractile function: (i) maximum delay in time-to-peak strain across the standard LV segments, (ii) standard deviation of time-to-peak values across standard LV segments; and, (iii) a novel vector-based methodology. The first two methods do not completely capture variation in strain curve morphology (**Figures S3A** and **S3B** in **Supporting Information S1**) and, as expected, demonstrated high intra- and inter-group variability at baseline (**Figures S3C** and **S3D**, respectively, in **Supporting Information S1**). In contrast, use of a new vector-based method resulted in more reliable measures within and between groups; segmental synchrony, assessed using this vectors-based method, was distinctly more variable in the AAC animals at 7 weeks compared to the sham animals (**Figure 2**). Measurements of dyssynchrony in the AAC animals mirrored the qualitative observations derived from topographical mapping. Furthermore, imaging-based evidence of dyssynchronous contractile function in the AAC animals was consistent with histologic evidence of regional fibrotic heterogeneity seen across the LV (**Figure 3**).

## Discussion

To our knowledge, this paper describes the first application of echocardiographic speckle-tracking based strain analyses for cardiac phenotyping in a mouse model of both pressure overload and reversal. We observed that contractile function, as reflected by systolic strain, was significantly reduced in response to increased pressure loading even early in the course of cardiac hypertrophy and then reversed in response to pressure relief. Importantly, these changes in strain were identified prior to detectable alterations in conventional measures of LV function such as fractional shortening. Furthermore, early changes in myocardial contractility were observed exclusively in the basal segments of the LV, suggesting that afterload stress induces regional cardiac dysfunction. In addition, early LV response to pressure overload involved not only changes in contractile function but also synchronicity of ventricular contraction. Notably, these changes in LV segmental synchrony were detected using a newly developed vector-based measure and were also more pronounced in the basal compared to apical regions.

### Regional Cardiac Dysfunction Induced By Pressure Overload

Whereas prior studies have demonstrated that global cardiac dysfunction is induced by afterload stress, [9] there has been sparse data on the effects of afterload stress on regional cardiac performance and its potential for reversibility. Regional variation in cardiac dysfunction is widely held as a pattern of cardiomyopathy more typically observed in the setting of ischemia and much less so in the setting of hypertension or other forms of global pressure overload. However, recent clinical studies of cardiac function utilizing echocardiographic measures of strain have reported relative differences in contractility of the basal versus apical LV segments in hypertensive patients compared to non-hypertensive. [2] The extent to which this regional variation in dysfunction is reversible has not previously been investigated. Our experimental data not only demonstrate the development of pronounced basal myocardial dysfunction in response to pressure overload, but also reversal of this dysfunction following reversal of pressure overload. Thus, such regional alterations in cardiac performance could serve as an early metric of the cardiac response to afterload stress and, in turn, the efficacy of therapeutic interventions designed to alleviate this stress.

The pathophysiology underlying the association between afterload stress and regional cardiac dysfunction is unknown. Notably, the basal myocardium undergoes the most vigorous change in displacement and, in turn, the highest velocity motion under normal conditions. [10] Accordingly, reduced basal tissue velocity is often seen in patients with hypertension as well as in patients with non-hypertensive cardiomyopathies.[11]–[13] Thus, the basal portions of myocardium appear to be particularly susceptible to early decrements in cardiac performance assessed by several measures and in response to different types of stress. Interestingly, a predilection for basal versus apical dysfunction has been observed in studies of aortic regurgitation [14] and dilated non-ischemic cardiomyopathy. [15] Additional research is needed to further investigate the predilection for basal versus apical dysfunction in response to various cardiac stressors that are thought to exert relatively global effects.

### Dyssynchrony Induced by Pressure Overload

If pressure overload stress is able to induce regional cardiac dysfunction, it then follows that this same stress may also lead to dyssynchronous segmental contraction. Indeed, we observed not only reduced basal contractility but also LV dyssynchrony following aortic banding and prior to any significant changes in overt fractional shortening. The tendency for subclinical LV dyssynchrony to arise in pressure overload states such as hypertension, but in the absence of overt cardiac failure, is a novel concept that has been only recently been suggested in the literature.[16]–[18] Our findings provide further support for the hypothesis that increased afterload induces variable stress across regions of the heart, rather than a uniform global stress, causing dyssynchronous contractility. Similar to measures of regional strain, measures of dyssynchrony may serve as markers of early myocardial dysfunction in the setting of pressure overload and, additionally, as markers of response to therapies design to relieve the effects of such overload.

It should be noted that although dyssynchrony has been assessed using a variety of strain-based imaging methods in humans, [3] these methods have limited utility in small animals. Thus, we employed a newly developed vector-based measure of dyssynchrony that proved illustrative in a murine model of pressure overload. Conventional measures of dyssynchrony are limited in mice due to 2 main technical reasons. First, the temporal resolution of echocardiographic imaging is limited in mice due to fast heart rates ranging from 600–700 beats per minute. Such rapid heart rates, along with a temporal resolution of approximately 230 frames per second, provide a frame time of 4.7 msec; thus, any detectable differences between groups and standard deviations are in the range of a single frame. Second, both the maximum range and standard deviation of time-to-peak strain values is based on the assumption that strain curves with differently timed peaks do not have a large amount of overlapping morphology; however, curve morphology can often overlap, particularly in the setting of high heart rates (**Figure S3** in **Supporting Information S1**). In contrast, the vector-based method described here for assessing segmental synchrony of contractile function across multiple myocardial regions accounts for both variation in morphology and time-to-peak strain values.

### Limitations

Speckle-tracking-based strain analyses can vary based on the echocardiographic views obtained for image acquisition, especially in short-axis views. Thus, our analyses were based primarily on data acquired from long-axis views. In particular, we acquired data from the parasternal long-axis view, which offers the advantage of using anatomical landmarks to minimize angular variation in mice. Because apical 4-chamber views are difficult to obtain in mice, parasternal views are preferred for long axis measurements. Although investigator-performed tracings are an additional source of variability, we have previously demonstrated excellent inter- and intra-observer variability in our laboratory through the use of a detailed standardized protocol. [4] The applicability and generalizability of the findings described in this report may be limited to the use of similar image acquisition equipment and image analysis software. However, ongoing advances in the field are likely to broaden the availability of strain-based analyses and promote further standardization of techniques.

### Conclusion

In a small animal model, we demonstrate evidence of dyssynchrony as well as regional myocardial dysfunction that was induced by pressure overload early with cardiac hypertrophy and attenuated with pressure relief. This regional pattern of dysfunction and dyssynchrony was detected non-invasively, using speckle-tracking-based strain analyses that allowed for serial comprehensive assessment of cardiac mechanical function. These findings illustrate how strain-based imaging can provide new insights into the character and pathophysiology of cardiac dysfunction resulting from cardiac injury or stress. Further research is needed to determine if the early alterations in myocardial performance, observed in this study, may be used as reliable markers of dysfunction in tests of genetic models and novel therapies designed to attenuate the development of hypertensive heart disease and heart failure.

## Supporting Information

Supporting Information S1 **The Supporting Information S1 file contains additional information relevant to the manuscript, including: Table S1 (conventional echocardiographic measures); Figure S1 (schematic overview of the anatomic LV regions seen in the parasternal long-axis view; Figure S2 (changes in global longitudinal strain and synchrony in a model of myocardial infarction); and, Figure S3 (conventional measures of dyssynchrony).**
(DOC)

Video S1 **Cine topographical mapping of strain throughout the cardiac cycle.** Topographical mapping of longitudinal (endocardial and epicardial) strain throughout the cardiac cycle is shown, where positive strain values are denoted by red (representing myocardial segmental expansion) and negative strain values are denoted by blue (representing myocardial segmental contraction). In sham animals (**A**), positive strain values (in red, denoting myocardial segmental expansion) appropriately appear only during end-diastole. In banded animals (**B**), positive strain values appear throughout the cardiac cycle, with positive endocardial strain occurring predominantly in the basal and mid regions of the LV compared to the apex. In de-AAC animals (**C**), positive strain values appropriately appear only during end-diastole, similar to sham animals. The video is available at: http://dump.tentacleriot.eu/SupplementalVideo.mp4.
(MP4)
