# Supplementary material for: Regional Cardiac Dysfunction and Dyssynchrony in a Murine Model of Afterload Stress
Source: PLoS One. 2013 Apr 1;8(4):e59915. doi: 10.1371/journal.pone.0059915 (PMC3613376; doi:10.1371/journal.pone.0059915)
Supplement: Supporting Information S1 — The Supporting Information S1 file contains additional information relevant to the manuscript, including: Table S1 (conventional echocardiographic measures); Figure S1 (schematic overview of the anatomic LV regions seen in the parasternal long-axis view; Figure S2 (changes in global longitudinal strain and synchrony in a model of myocardial infarction); and, Figure S3 (conventional measures of dyssynchrony). (DOC) [file pone.0059915.s001.doc]

**SUPPORTING INFORMATION**

**Supplemental Methods**

**Echocardiography**

For each echocardiography examination, animals were gently restrained on a heated platform. Warm ultrasound gel was placed on the depilated chest and B-Mode loops from parasternal long- and parasternal short-axis were obtained. For standard measurements of left ventricular (LV) function and geometry, M-mode images were recorded from parasternal short-axis images at the mid-papillary level. Fractional shortening and LV diameter were measured from M-mode images. Wall thickness is calculated as the average of anterior and posterior wall thickness in M-mode images. LV mass was calculated from parasternal long-axis tracings using a modified Simpson’s formula.

To ensure similar pressure gradient in animals subjected to ascending aortic constriction, gradients across the aortic stenosis were measured using continuous wave Doppler echocardiography. First, the ascending aorta was visualized from the left ventricular outflow tract to the branching of the innominate artery. After the stenosis was visualized in B-mode images, color Doppler was added to visualize blood flow. The pulse wave Doppler gate was placed at the position with the highest visible flow through the stenosis and pulse-wave curves were recorded. Next, the pulse-wave gate was placed in the mid-artery before the stenosis and flow was measured. The peak gradient through the stenosis was calculated as the difference of peak pressure through stenosis and peak pressure prior to the stenosis.

**Validation of Dyssynchrony Methods in a Model of Myocardial Infarction**

To validate and provide reference data for interpreting non-invasive measures of myocardial dyssynchrony in murine models of cardiac stress, we analyzed a data set obtained using speckle-tracking-based echocardiography in a previous published study of murine model of myocardial infarction (MI).[^1^](#_ENREF_1) In brief, a total of 16 mice were randomly assigned to permanent coronary ligation of the left anterior descending artery (N=6), coronary ligation followed by administration of an angiotensin converting enzyme inhibitor (N=5), or sham procedure (N=6). All mice underwent serial echocardiography with strain analyses. For the current study, we additionally performed post-hoc echocardiographic analyses of LV segmental synchrony using the same analytical technique described in the **Methods** section of the main manuscript.

**Supplemental Results**

**Dyssynchrony in Myocardial Infarction**

As we have previously described,[^1^](#_ENREF_1) progressive changes in myocardial performance were observed in all animals at serial time points following MI, with significant differences seen between the MI groups and the vehicle (sham) group of mice. Importantly, as reported previously,[^1^](#_ENREF_1) there was a significant difference in global longitudinal shortening seen at 3 weeks between the treated MI+ACEi mice and the untreated MI mice (**Supplemental Figure 2A**). With respect to measures of dyssynchrony, maximal delay of time-to-peak longitudinal strain was higher (worse) in the untreated MI group compared vehicle group at 3 weeks, but was not significantly different between the untreated MI group the treated MI+ACEi group (**Supplemental Figure 2B**). In contrast, standard deviation of time-to-peak longitudinal strain did not differentiate between MI and vehicle groups at 1 week, but was significantly different between the untreated MI and the treated MI+ACEi groups at 3 weeks (**Supplemental Figure 2C**). Finally, measures of longitudinal strain angle appropriately differentiated between MI and vehicle groups at 1 week following MI and, furthermore, also demonstrated greater dyssynchrony in the untreated MI compared to the treated MI+ACEi groups at 3 weeks (**Supplemental Figure 2D**). Thus, among the 3 methods tested for quantifying LV dyssynchrony, longitudinal strain angle demonstrated the greatest ability to detect both relative impairment and recovery of synchronous myocardial function in this murine model of MI. These findings paralleled the previously reported similar findings observed for global longitudinal strain.

**Supplemental References**

1. Bauer M, Cheng S, Jain M, Ngoy S, Theodoropoulos C, Trujillo A, Lin FC, Liao R. Echocardiographic speckle-tracking based strain imaging for rapid cardiovascular phenotyping in mice. *Circ Res*. 2011;108:908-916.

**Supplemental Figures Legends**

**Figure S1. Schematic overview of the anatomic LV regions seen in the parasternal long-axis view:** BA, basal anterior; MA, mid anterior; AA, apical anterior; BI, basal inferior; MI, mid inferior; AI, apical inferior.

**Figure S2.** **Changes in global longitudinal strain and synchrony in a model of myocardial infarction (MI).** Global peak longitudinal strain (endocardial) was significantly worse in the MI versus sham groups at 1 week, and also significantly improved in the ACE inhibitor treated MI group at 3 weeks (**A**). Maximal delay of time-to-peak longitudinal strain was higher (worse) in the untreated MI group compared sham group, but not significantly different from the treated MI group at 3 weeks (**B**). Standard deviation of time-to-peak longitudinal strain was significantly different between treated and untreated MI groups at 3 weeks, but did not differentiate between even MI and sham groups at 1 week (**C**). Longitudinal strain angle differentiated between MI and sham groups at 1 week, and also demonstrated greater dyssynchrony between the treated MI and the untreated MI groups at 3 weeks (**D**).

**Figure S3.** **Conventional measures of dyssynchrony.** Representative schematics are shown for detecting dyssynchrony based on difference in time-to-peak longitudinal strain values across LV regions (**A**) and on difference in mean vector angle (**B**). Dyssynchrony measured as the difference between the earliest and latest time-to-peak values (among 6 regional curves) are highly variable and not significantly different across the sham, banded, and de-AAC groups at baseline and at 7 weeks (**C**); similar findings were observed for dyssynchrony measured as the standard deviation of time-to-peak values for the 6 regional curves (**D**).

**Table S1.** Conventional echocardiographic measures.

|  | **Baseline** | | | **1 Week** | | | **7 Weeks** | | | **P value** |
| --- | --- | --- | --- | --- | --- | --- | --- | --- | --- | --- |
|  | Sham | AAC | de-AAC | Sham | AAC | de-AAC | Sham | AAC | de-AAC |
| Heart Rate (1/min) | 522±44 | 549±35 | 535±66 | 643±33 | 627±20 | 611±19 | 701±3 | 673±9 | 697±14 | 0.406 |
| 2D LV Dimension (mm) | 3.3±0.2 | 3.0±0.2 | 3.1±0.2 | 2.9±0.2 | 2.7±0.2 | 2.8±0.1 | 3.0±0.1 | 2.9±0.2 | 3.0±0.1 | 0.029 |
| LV FS (%) | 36.2±3.0 | 39.9±5.7 | 37.9±3.1 | 46.3±3.1 | 38.6±4.2 | 36.7±4.7 | 52.9±1.2 | 44.3±3.8 | 42.1±2.6 | 0.031 |
| LV Mass (mg) | 107.3±5.2 | 102.6±5.5 | 108.3±4.9 | 103.4±2.1 | 146.1±11.4* | 146.4±6.8* | 96.7±6.1 | 141.8±12.3* | 120.2±8.4 | <0.001 |
| LV Wall Thickness (mm) | 0.9±0.1 | 0.9±0.1 | 0.9±0.0 | 1±0.0 | 1.3±0.1* | 1.3±0.0* | 1.1±0.0 | 1.3±0.1* | 1.1±0.0† | <0.001 |
| Heart Weight (mg) |  | | | | | | 174±0.9 | 220±1.3 | 178±13.2 | <0.05 |

LV, Left Ventricular.

Main P values shown are from ANOVA tests of between-group differences.

*P<0.05 for post-hoc t-test comparison versus sham.

†P<0.05 for post-hoc t-test comparison versus AAC.

**Figure S1.**

**
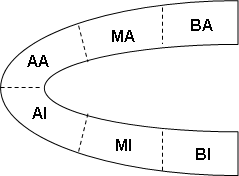
**

**Figure S2.**

**
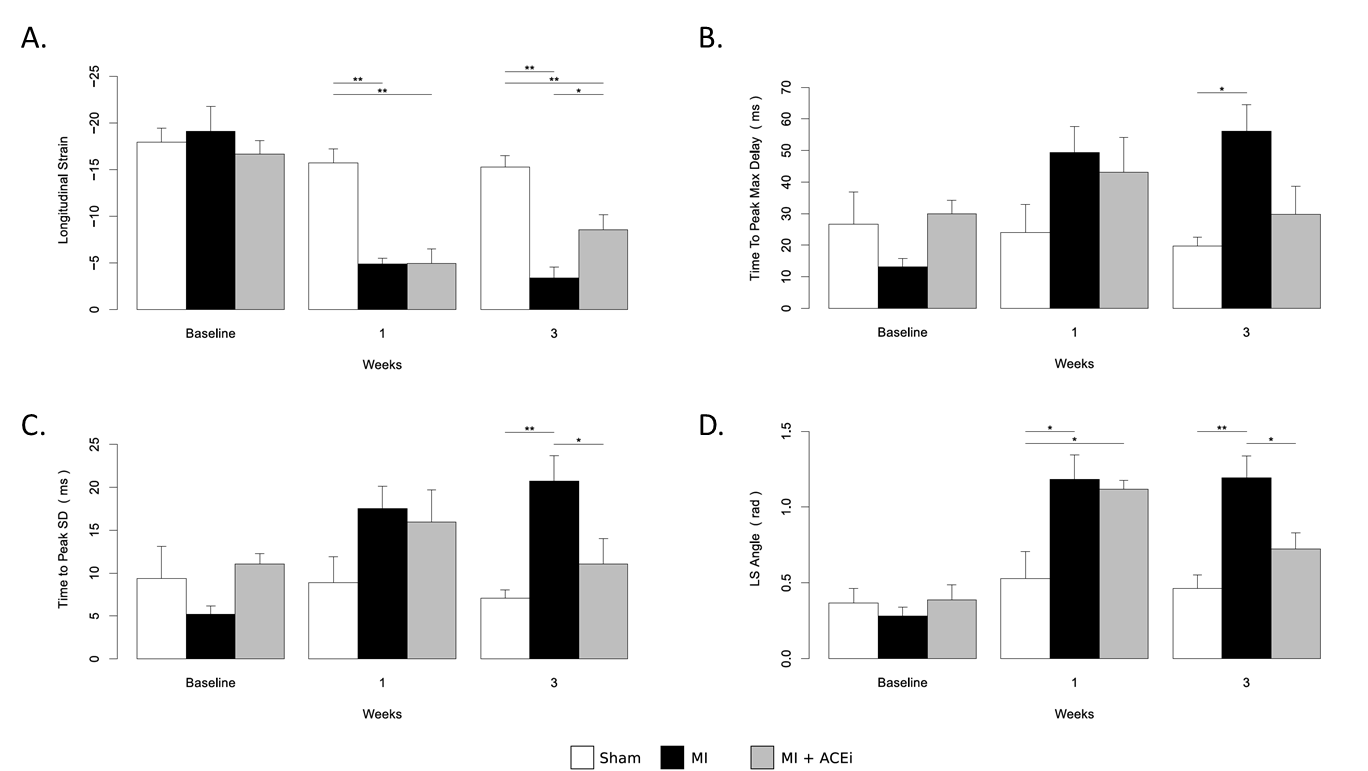
**

**Figure S3.**

Sham

AAC

de-AAC
